# Exploring the Antibacterial Potential of Green-Synthesized MgO and ZnO Nanoparticles from Two Plant Root Extracts

**DOI:** 10.3390/nano13172425

**Published:** 2023-08-26

**Authors:** Bachir Ben Seghir, Meriem Hima, Fatima Moulatti, Ibtihal Sahraoui, Ilham Ben Amor, Soumeia Zeghoud, Hadia Hemmami, Imane Kouadri, Asma Ben Amor, Mohammed Messaoudi, Shakeel Ahmed, Abdelkrim Rebiai, Pawel Pohl

**Affiliations:** 1 Department of Process Engineering and Petrochemical, Faculty of Technology, University of El Oued, El Oued 39000, Algeria; meriem.hima@gmail.com (M.H.); fatima.moulatti@gmail.com (F.M.); ibtihal.sahraoui@gmail.com (I.S.); ilhambenamor97@gmail.com (I.B.A.); zsoumeia@gmail.com (S.Z.); hemmami.h@gmail.com (H.H.); benamor.asma39@gmail.com (A.B.A.); 2 Renewable Energy Development Unit in Arid Zones (UDERZA), University of El Oued, El Oued 39000, Algeria; kouadri-2013@hotmail.fr (I.K.); rebiai-abdelkrim@univ-eloued.dz (A.R.); 3 Laboratory of Industrial Analysis and Materials Engineering (LAGIM), University of 8 May 1945, P.O. Box 401, Guelma 24000, Algeria; 4 Department of Process Engineering, Faculty of Science and Technology, University of 8 May 1945, P.O. Box 401, Guelma 24000, Algeria; 5 Nuclear Research Centre of Birine, P.O. Box 180, Ain Oussera 17200, Algeria; messaoudi2006@yahoo.fr; 6 Laboratory of Applied Chemistry and Environment (LCAE), Department of Chemistry, Faculty of Exact Sciences, University of Hamma Lakhdar El Oued, B.P. 789, El Oued 39000, Algeria; 7 Department of Chemistry, Government Degree College Mendhar, Kashmir 185211, India; shakeelchem11@gmail.com; 8 Higher Education Department, Government of Jammu and Kashmir, Srinagar 190001, India; 9 University Center for Research and Development (UCRD), Chandigarh University, Mohali 140413, India; 10 Department of Analytical Chemistry and Chemical Metallurgy, Faculty of Chemistry, University of Science and Technology, Wyspianskiego 27, 50-370 Wroclaw, Poland

**Keywords:** green synthesis, nanoparticles, antibacterial, inhibition, bacterial activity

## Abstract

The green approach-based nanoparticle synthesis is considered a more cost-effective and ecologically responsible method of producing nanoparticles than other standard techniques. A major accomplishment in resolving these issues is the use of nanoparticles for environmental pollution remediation. This article describes a simple method for producing MgO and ZnO nanoparticles (NPs) using aqueous extracts of *Zingiber officinale* and *Glycyrrhiza* roots as the stabilizing and reducing agents, respectively. Fourier transform infrared spectroscopy (FTIR), X-ray diffraction (XRD), scanning electron microscopy (SEM), and energy-dispersed X-ray (EDX) spectroscopy methods were used to characterize the biologically synthesized metal oxide nanoparticles (MO NPs). The XRD results showed that the mean crystallite sizes of synthesized ZnO and MgO NPs, which have excellent purity, are 12.35 nm and 4.83 nm, respectively. The spherical or elliptical shape of the synthesized NPs was confirmed by the SEM analysis. The antibacterial activity of the synthesized NPs against both Gram-negative and Gram-positive bacteria was thoroughly investigated. With a medium zone of inhibition of 7 to 10 mm, the as-synthesized MgO NPs and ZnO NPs demonstrated moderate antibacterial activity towards various bacterial strains.

## 1. Introduction

Nanotechnology is now considered a verified technology with various uses in the chemical, pharmaceutical, mechanical, and food processing fields. Applications of nanotechnology in computers, energy production, optics, medicinal delivery, and environmental sciences are particularly fascinating [1,2]. To advance the distinctive and adaptable properties of the used materials, researchers have developed excitingly new materials at the nanoparticle scale (with sizes ranging from 1 to 100 nm). Their applications in science and technology are numerous [3]. Due to their incredibly small size and high surface-to-volume ratio, nanoparticles (NPs) have drawn a lot of attention because they significantly differ from masses of the same element in terms of their physical, biological, catalytic, thermal, and electrical properties [4].

Organometallic synthesis, sol–gel processing, spray pyrolysis, homogeneous precipitation, thermal evaporation, mechano-chemical synthesis, mechanical milling, and microwave-assisted synthesis methods are just a few examples that have been suggested for the synthesis of NPs [3]. However, they generally pose a threat to the environment [5]. An alternative to these methods that is simple to control and design is green NP synthesis [6]. The conventional methods for the synthesis of NPs have a number of disadvantages, including lengthy processing, high costs, and, in particular, the necessity to use toxic compounds. Due to these restrictions, the majority of pertinent studies have focused on quick and environmentally friendly protocols for the synthesis of various NPs [7,8]. The development of environmentally friendly processes for synthesizing nanoscale materials has recently received a lot of attention from material scientists. In this regard, the green synthesis of NPs, particularly when using plant extracts, is a very promising trend that is regarded as straightforward, affordable, and non-toxic in nature [8,9,10]. Therefore, scientists put a lot of effort into the new method of synthesis of NPs using environmentally friendly means, including the application of plant leaf extracts, fruit peel extracts, algae extracts, etc., as reducing and stabilizing factors. Successful methods for the synthesis of some non-toxic, inexpensive, and environmentally friendly nanoparticles have been developed in the past [3,11,12,13,14,15,16].

MgO and ZnO NPs are useful metal oxide (MO) materials with a variety of properties, including high chemical stability and photostability, a wide band gap, and a low dielectric constant. These properties have been used so far in numerous industrial and scientific applications, including atalysis and the development of antimicrobial and antibacterial materials. Because of this, it is important to develop new methods of the green synthesis of MgO NPs and ZnO NPs that would be helpful for the degradation of hazardous organic contaminants as well as exhibit certain antibacterial activities [17,18,19,20].

The use of nanoparticles (NPs) in basic research and practical applications is widespread. Nanoparticle (NP) application has been shown to both promote and inhibit the seedling emergence and growth rates of diverse plant species. The effect of the NPs is determined by their chemical composition, particle size and shape, and the method used for their synthesis. These tiny particles with a high surface area and sizes ranging from 1 to 100 nm are unique and versatile in a wide spectrum of applications [21,22].

The objective of this study was to synthesize MgO NPs with the *Zingiber officinale* extract and ZnO NPs with the *Glycyrrhiza* extract. Then, we aimed to characterize them and assess their antibacterial activity against various bacterial strains that represent Gram-positive and Gram-negative human pathogenic microorganisms.

## 2. Materials and Methods

### 2.1. Chemicals

Chemicals including magnesium chloride hexahydrate (MgCl_2_·6H_2_O; 99.9%), zinc chloride hexahydrate (ZnCl_2_·6H_2_O; 99.9%), sodium hydroxide (NaOH; 99.9%), and dimethyl sulfoxide (DMSO, 99%) were acquired from BIOCHEM Chemopharma (Cosne-Cours-sur-Loire, France). The Mueller–Hinton agar was purchased from Bioscan Industrie, Ouled Sabor, Algeria. For the experimental work, de-ionized water was used throughout all the experiments.

### 2.2. Preparation of Aqueous Extracts

The roots of *Zingiber officinale* and *Glycyrrhiza* were bought in a local market in the Walleye El Oued region. To prepare the extract, the plant parts were put in an electric grinder and turned into a fine powder. A total of 100 g of this plant powder were mixed with 600 mL of distilled water and heated at 100 °C for 60 min. The resulting extract was filtered through Whatman filter paper No. 1, and then it was stored in a dry place at 4 °C.

### 2.3. Green Synthesis of MgOand ZnO Nanoparticles

**Figure 1:**
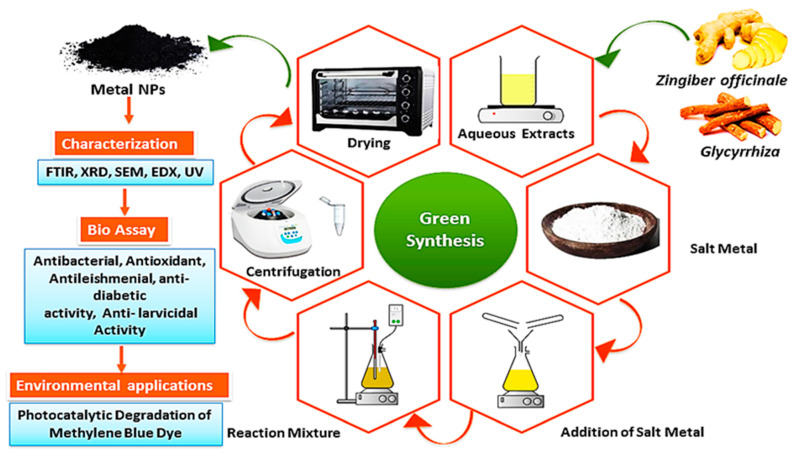
The schematic representation of the synthesis of MgO NPs and ZnO NPs using the root extracts.

A total of 5 mL of the prepared plant extract from both botanical extracts was placed separately. Then, 10 mL of the solution of MgCl_2_ for the MgO synthesis from Zingiber extract and ZnCl_2_ for the ZnO synthesis from Glycyrrhiza extract at a concentration of 0.1 mol/L was added to 50 mL of de-ionized water and magnetically stirred for the green. After that, a 1 mol/L NaOH solution was slowly added until the solution reached a pH of 8. This step is crucial to obtaining an alkaline medium that allows for and facilitates the process of green synthesis. The mixture was continuously stirred at 80 °C for 6 h. Within 30 min from that, yellowish products, being MgO NPs and ZnO NPs precipitates, were formed. The solutions were then centrifuged at 6000 rpm for 10 min, washed well several times, and the product was annealed at 450 °C for 3 h. The resulting samples were collected and stored in a desiccator for the characterization of the nanomaterials and the investigation of their antibacterial properties [23,24]. Figure 1 shows the schematic diagram of the preparation of the MgO and ZnO NPs.

### 2.4. Characterization Methods

Using a JASCO V-670 UV–visible spectroscopy instrument, Tokyo city, Japan with a diffuse reflectance attachment for powder samples, the absorption spectra of the biosynthesized MgO NPs and ZnO NPs were acquired within the spectral range of 200–800 nm. Using Fourier transform infrared spectroscopy (FTIR), the biosynthesized MgO NPs and ZnO NPs were also analyzed. A Perkin Elmer Spectrum 1000 instrument, Waltham City, Massachusetts, USA was used in the attenuated total reflection mode, while the FTIR spectra were acquired within the spectral range of 4000–400 cm^−1^ and with a resolution of 4 cm^−1^. With the aid of energy dispersive X-ray spectroscopy (EDS) and field emission scanning electron microscopy (FE-SEM), the morphology and composition of the biosynthesized MgO and ZnO NPs were examined (JEOL-JSM 6500F, JEOL, Tokyo, Japan). The biosynthesized MgO NPs and ZnO NPs were also examined using high-resolution transmission electron microscopy (HRTEM, Tecnai F20 G2, Philips, Amsterdam, The Netherlands) at an accelerating voltage of 200 kV. In the latter case, the shape, particle size, and crystallinity of both nanomaterials were examined. Using an X-ray diffractometer (XRD-7000, Shimadzu Co., Kyoto, Japan) fitted with a Cu target to provide the CuK radiation (λ = 1.54056 Å at 40 kV and 30 mA), the crystal structure and average crystalline size of the synthesized MgO NPs and ZnO NPs were characterized. The XRD spectra acquired for the 2θ angles ranged from 10° to 80°.

The crystallite size (D) of the biosynthesized nanomaterials was calculated using Scherrer’s Equation (1):

(1) $$D=\frac{K\;\lambda }{\beta \cos\theta }$$

where *K* = 0.94 is the Scherrer’s constant, *λ* is the X-ray wavelength, *θ* is the Bragg’s diffraction angle, and *β* is the peak width of the diffraction line at half width at its maximum intensity.

### 2.5. Antibacterial Activity

The agar well diffusion method [25] was used to test the antibacterial activity of MgO NPs and ZnO NPs against the bacterial strains of *Staphylococcus aureus* (ATCC6538), *Bacillus subtilis* (ATCC6633), *Listeria innocua* (CLIP74915), *Pseudomonas aeruginosa* (ATCC9027), and *Salmonella typhimurium* (ATCC14028). The prepared nutrient agar plates were swabbed with 100 µL of the 24 h matured bacterial strain broth culture using sterile rods. Each Petri plate had a 6 mm well that was created using a sterile cork borer. Solutions of ZnO NPs and MgO NPs containing 2, 4, and 6 mg/mL were used to study the antibacterial activities of these nanomaterials. A 10% (*v*/*v*) DMSO solution was used to prepare the solutions of both nanomaterials, which were then added using sterile micropipettes to the wells.

## 3. Results and Discussion

In Figure 1, a visual representation of the green synthesis of MgO NPs and ZnO NPs, as well as their characterization in this study, was presented. The results showed that both the MgO NPs and the ZnO NPs were successfully generated after a variety of analytical techniques were utilized to characterize these NPs.

### 3.1. UV–Vis Spectral Analysis

**Figure 2:**
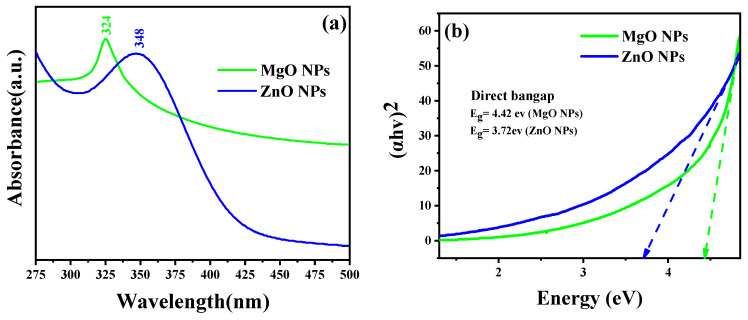
(**a**) UV–Vis diffuse reflectance spectra and (**b**) the Tauc plot for the band gap analysis.

As the absorption bands of simples are related to the diameter and aspect ratio of metallic NPs, UV–Vis absorption spectroscopy is the most useful technique for characterizing their optical characteristics and electronic structure [26]. In this study, the prepared MO NPs were confirmed by UV–Vis spectroscopy. Radiation with wavelengths between 300 and 800 nm is commonly used to characterize various metallic NPs with sizes between 2 and 100 nm [27]. The UV–Vis spectra of MgO NPs and ZnO NPs were acquired for the 250–800 nm spectral range, as shown in Figure 2. The prepared samples’ colloidal solutions were kept at 25 °C for 24 h. The ZnO sample clearly demonstrated that the maximum absorption peak appeared at 348 nm, which is reported for ZnO NPs with the hexagonal wurtzite structure [25]. The UV–Vis spectrum of the MgO sample also displayed a single absorption band at 324 nm. Based on Equation (2), it was determined that the optical band gap energies of the ZnO NPs and MgO NPs at the maximum absorption peaks were 3.72 eV and 4.42 eV, respectively.

These findings confirmed that both oxides could be activated by the influence of UV light.

(2) Eg=hv/λ=1240/λ

where v is the wavelength of the maximum absorption, h is Planck’s constant, and c is the speed of light. In addition to the optical band gap at the wavelength of maximum absorption, the band gap of the synthesized ZnO NPs and MgO NPs was also estimated based on the Tauc equation and the Tauc plot formula shown in Equation (3) and Figure 2b, respectively. According to the Tauc equation, the photon energy (*hυ*) is related to the optical band gap energy (*E_g_*) of a semiconductor (*αhυ*)^2^ by the following relationship:

(3) $${\left(\alpha hv\right)}^{2}=D(hv-E_{g})$$

where α and *D* are the absorption coefficient and the optical transition-dependent constant of the material under investigation, respectively. *hv* is the photon’s energy from the incident radiation, and *E_g_* is the band gap of the synthesized material, while the exponent “n” indicates the nature of the optical transition in the semiconductor. Figure 2b shows the linear extrapolation of the (*αhv*)^2^ vs. *hv* plots provided by the band gap (*E_g_*) values for the synthesized NPs. The higher value of the bandgap compared to that of the bulk is due to the quantum confinement effect.

### 3.2. FTIR Spectra

**Figure 3:**
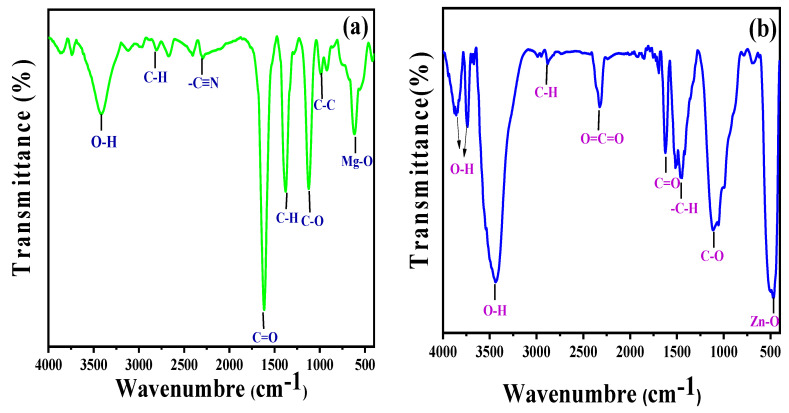
FTIR spectra of MgO NPs synthesized by ginger (*Zingiber officinale Roscoe*) root extract (**a**) and ZnO NPs synthesized by *Glycyrrhiza root extract* (**b**).

Figure 3a,b displays the FTIR spectra of *Zingiber officinale* and ginger root-derived MgO and ZnO NPs. The broad band that is visible between 3850 and 3431 cm^−1^ likely corresponds to OH [28,29]. The distinct absorption band at 2325 cm^−1^ denoted the CO_2_ stretching vibrations, either aerial or CO_2,_ within the NP grains. The rapid sorption of atmospheric CO_2_ indicated a greater surface area of the synthesized materials [30]. The sharp peaks at 1392 and 1064 cm^−1^ corresponded to the stretching vibrations of the C–H aliphatic amines, while the broad band at 1622–1613 cm^−1^ corresponded to the C=O aromatic ring stretching vibrations [31]. The intense peaks at 612 cm^−1^ and 473 cm^−1^ confirmed the stretching vibrations within MgO NPs and ZnO NPs, respectively [29]. The peak shifts of MgO NPs and ZnO NPs in this range (2796–2876, 1622–1610, 1455–1380 cm^−1,^ and 1110 cm^−1^) after the bioreduction are indicative of phytochemicals, terpenoids, flavonoids, polyols, and proteins. These compounds possess functional groups such as ketones, alcohols, carboxylic acids, and amines that act as chelating and capping agents during bioreduction. The N–H, O–H, and H-bonded phenols and alcohol stretching oscillations of amide groups were represented because of the powerful and broad peak at 1500–2000 cm^−1^, which were previously reported in the literature [25,29,31,32,33,34].

### 3.3. X-ray Powder Diffractogram

**Figure 4:**
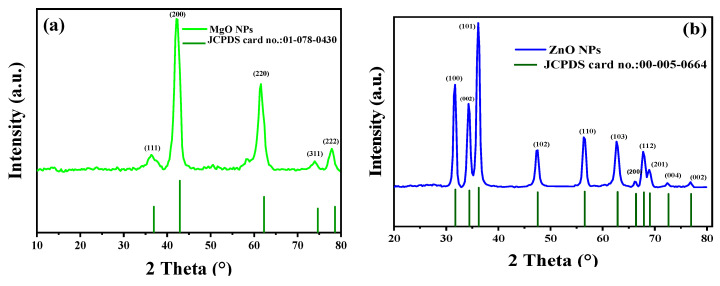
XRD patterns of synthesized MgO NPs by the *ginger* (*Zingiber officinale Roscoe*) root extract (**a**) and synthesized ZnO NPs by the *Glycyrrhiza* root extract (**b**).

The MgO and ZnO NPs’ phases and crystal structures were described using XRD analysis. As shown in Figure 4, the XRD pattern of MgO NPs revealed five significant peaks at 36.51°, 42.10°, 61.51°, 74.02°, and 77.95°, which, respectively, represent the (111), (200), (220), and (311) planes. The XRD pattern revealed that the synthesized MgO NPs were cubic in structure and crystalline in nature, according to the Joint Committee on Powder Diffraction Standards (JCPDS file no. 01-078-0430) [35]. It was also noted that the last three peaks could be indexed to the hexagonal crystal structure of MgO NPs. The crystal size was calculated using Scherrer’s formula, with the particle size around 4.83 nm. Almost similar XRD results were reported for MgO NPs prepared by other biogenic materials [35,36,37,38,39,40].

Additionally, the crystalline structure of green synthesized ZnO NPs was investigated using XRD analysis. The peaks identified at 2θ = 31.7°, 34.35°, 36.18°, 47.41°, 56.50°, 62.62°, 66.20°, 68.95°, 72.62°, and 76.91° corresponded to the (100), (002), (101), (102), (110), (103), (200), (112), (201), (004), and (002) planes, respectively. These peaks were evidence of the hexagonal wurtzite structure of the ZnO NPs [35,36,37]. The polycrystalline nature of the ZnO NPs, revealed by the observed XRD pattern, was univocally verified using JCPDS file no. 00-055-0664 [41]. In addition, Ali et al. obtained a comparable XRD pattern for pure ZnO NPs and various C-doping concentrations in ZnO NPs [42]. According to the calculations made, the average crystallite size was 42.50 nm. The lack of any XRD peaks other than those of ZnO NPs further demonstrated that the synthesized nanopowders were free of impurities.

### 3.4. Scanning Electron Microscopy–Energy-Dispersive X-ray (SEM–EDX)

**Figure 5:**
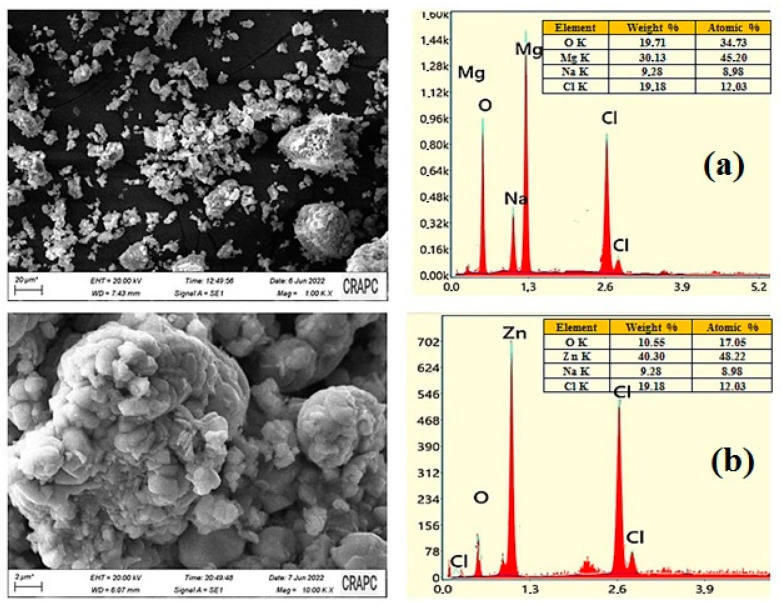
The results of the SEM–EDX analysis and the particle size distributions of MgO NPs (**a**) and ZnO NPs (**b**).

The SEM–EDX examination is widely regarded as essential to studying the morphological homogeneity and chemical composition of biosynthesized materials. The SEM micrographs and the results of the EDX analysis of the MgO and ZnO NPs, synthesized under optimal conditions, are shown in Figure 5a and Figure 5b, respectively.

Figure 5 exhibits a typical SEM image of the MgO NPS sample, indicating that the synthesized material possessed irregular particle morphology, a quasi-spherical-like shape, and was rather dense and agglomerated. The purity of MgO NPs was also analyzed using SEM–EDX. The characteristic peaks for Mg and O were only identified in the spectrum provided by the EDX analysis, based in Figure 5, it is showning these findings support the conclusion that MgO NPs of high purity were formed. Indeed, the percent weight of Mg and O in the green synthesized MgO NPs was found to be 57.25% and 38.99%, respectively. No other peak in the spectrum was found that suggested that the fabricated MgO nanostructures were pure and contained no other impurities.

The SEM image illustrates that ZnO NPs were predominantly spherical in shape and aggregated into larger particles and small aggregates. The EDX analysis of ZnO NPs was carried out to determine their elemental composition, as presented in Figure 5b. Based on the EDX spectrum, where the sharp peaks for Zn and O were identified, 70.30% (Zn) and 28.55% (O) percentage weights were established for both elements. These indicated the formation of pure ZnO NPs.

### 3.5. Antibacterial Properties

The antibacterial activity of MgO NPs and ZnO NPs was tested against Gram-positive bacteria (*Staphylococcus aureus*, *Bacillus subtilis*, *Listeria innocua*) and Gram-negative bacteria (*Pseudomonas aeruginosa*, *Salmonella typhimurium*).

The culture plates were prepared using a sterile glass rod and streaked with 100 L of a 24 h matured broth culture of the respective bacterial strains. Each Petri plate had six 6 mm wells that were created with a sterile cork borer. MgO NPs and ZnO NPs were suspended and present in DMSO at various concentrations (2, 4, and 6 mg/mL). Each experiment was performed in triplicate. Table 1 provides a summary of the diameters of the inhibition zones following the incubation period. Both ZnO NPs and MgO NPs were established to exhibit excellent antibacterial activity against the examined bacterial strains except for *Staphylococcus aureus*. The lowest and highest inhibition zones were observed against *Salmonella typhimurium* (10 ± 0.19 mm) and *Bacillus subtilis* (17 ± 0.35 mm), respectively. However, the assessed antibacterial activity was found to vary with the concentration of NPs. In general, the upsurge of multidrug-resistant bacterial strains provoked a need for the development and advancement of a new group of potent antibacterial agents. ZnO NPs are generally considered to be stable and safe antimicrobial agents, as they practically do not harm humans or animals. The antibacterial action of these NPs results from the release of reactive oxygen species (ROS) from the surface of ZnO NPs. This causes oxidative stress by damaging the cell membranes, cellular proteins, and DNA when in contact with the bacterial cells and accumulating in their microorganisms. These NPs release antimicrobial ions (Zn^2+^) inside the cells, causing a toxic microenvironment and finally damaging the membrane and intracellular area.

There are differences between MgO NPs and ZnO NPs in terms of their antibacterial effects. Generally, zinc oxide is considered more effective in killing bacteria compared to magnesium oxide [6,8,37]. This is attributed to the ability of zinc oxide to release zinc ions, which are toxic to bacteria. These ions can interact with bacterial cell membranes, disrupt cellular processes, and cause cell death.

Green synthesis using natural plant extracts like *Zingiber officinale* and *Glycyrrhiza* roots offers numerous advantages. It eliminates harmful chemicals, reduces environmental impact, and is cost-effective due to the affordability and availability of plant extracts. This method promotes sustainability by utilizing renewable resources and is biocompatible and safe for applications in biomedical and pharmaceutical fields. Additionally, it has the potential to enhance the therapeutic properties of the synthesized nanoparticles. However, there are challenges associated with green synthesis. The composition of plant extracts can vary, leading to variations in the properties of the nanoparticles. Limited control over nanoparticle characteristics can affect reproducibility and optimization. Green synthesis may yield lower quantities or slower reaction rates compared to conventional methods, posing challenges for large-scale production. Batch-to-batch variation is also a possibility, affecting the consistency and reliability of the synthesized nanoparticles.

## 4. Conclusions

In the current study, MgO and ZnO NPs were simply and sustainably synthesized from the aqueous extracts of the roots of Zingiber officinale and Glycyrrhiza, respectively. Moreover, the products of MgO and ZnO NPs were characterized by XRD, demonstrating that they possessed hexagonal wurtzite and face-centered cubic structures, respectively. In addition, the NPs MgO and ZnO were characterized for their morphology and chemical content, as well as the functional groups that capped and stabilized their structures. The size of these nanomaterials was also determined using SEM, UV–Vis, and FTIR spectroscopy. According to the data obtained from MgO and ZnO NPs in this study, their sizes were determined to be 12.4 nm and 4.8 nm, respectively, with both NPs exhibiting spherical-like morphologies. Moreover, the NPs of MgO and ZnO exhibit antibacterial efficacy against Gram-positive bacteria (*Staphylococcus aureus*, *Bacillus subtilis*, *Listeria innocua*) and Gram-negative bacteria (*Pseudomonas aeruginosa*, *Salmonella Typhimurium*) bacterial strains, as revealed by moderate inhibition zones.

## Figures and Tables

**Table 1:** Zone of inhibition synthesized of MgO and ZnO nanoparticles.

Concentration (mg⁄mL)		*Salmonella typhimurium*	*Staphylococcus aureus*	*Listeria innocua*	*Bacillus subtiliis*	*Pseudomonas aeruginosa*
		Zone of Inhibition (mm)				
ZnO	2	0	0	13 ± 0.15	17 ± 0.35	12 ± 0.14
	4	10 ± 0.19	0	11 ± 0.45	13 ± 0.7	13 ± 0.12
	6	0	0	12 ± 0.30	15 ± 0.17	15 ± 0.11
MgO	2	0	0	0	0	10 ± 0.25
	4	07 ± 0.30	0	0	0	0
	6	0	0	06 ± 0.30	0	0

## Data Availability

Data are available in the manuscript.
